# The Prevalence of Celiac Disease-Specific Auto-Antibodies in Type 1 Diabetes in a Moroccan Population

**DOI:** 10.1155/2019/7895207

**Published:** 2019-09-19

**Authors:** Ider Oujamaa, Majda Sebbani, Lahcen Elmoumou, Aïcha Bourrahouate, Rabiy El Qadiry, Soufian El Moussaoui, Imane Ait Sab, Mohamed Sbihi, Laila Ennazk, Ghizlane El Mghari-Tabib, Nawal El Ansari, Hicham Baizri, Mohamed Amine, Brahim Admou

**Affiliations:** ^1^Laboratory of Immunology, University Hospital of Marrakech, Marrakech, Morocco; ^2^Department of Public Health and Epidemiology, PCIM Research Laboratory, Cadi Ayyad University, Marrakech, Morocco; ^3^Department of Pediatrics, University Hospital of Marrakech, Marrakech, Morocco; ^4^Department of Endocrinology, University Hospital of Marrakech, Marrakech, Morocco; ^5^Department of Endocrinology, Ibn Sina Military Hospital, Marrakech, Morocco; ^6^ERCIM Research Team, Faculty of Medecine and Pharmacy, Cadi Ayyad University, Marrakech, Morocco

## Abstract

**Objective:**

We aimed to determine the prevalence of specific auto-antibodies to celiac disease (CD) in Moroccan type 1 diabetic (T1D) patients and compare the clinical and biological characteristics of seropositive and seronegative cases.

**Patients and Methods:**

A cross-sectional study was carried out on 276 T1D patients including 109 adults and 167 pediatric cases. The screening for CD was performed by an Elisa IgA anti-tissue transglutaminase antibody (tTGA) testing, combined with IgA quantification by nephelometry. Positive-IgA-tTGA cases were secondly tested for anti-endomysial antibodies (EMA) using an immunofluorescence technique, and the IgA deficiency cases were screened for IgG-tTGA. Patients with low positive tTGA titers underwent HLA-DQ2/DQ8 typing. Sociodemographic and clinical data of the patients were collected using a hetero-administered questionnaire. The comparison of clinical and biological data between seropositive and seronegative diabetics was done using independent *T*, Mann–Whitney *U*, chi-squared, and Fisher tests, which were considered significant if *p* value <0.05.

**Results:**

The prevalence of CD-specific auto-antibodies was estimated to be 9.1% (IC = 95%), with 25 positive cases in tTGA and EMA testing. Eight cases displayed low titers of IgA-tTGA, among which 4 were positive for HLA-DQ2, 1 for HLA-DQ8, and 1 for both DQ2 and DQ8. The other 2 cases had a biopsy-proven CD. Compared to seronegative patients, seropositive cases had a higher percentage of associated autoimmune disorders (16% vs. 2.4%, *p*=0.008), with a significant lower height Z-scores (median: −0.90 (−3.93 to 0.95) vs. −0.51 (−4.54 to 2.18), *p*=0.029) and a higher HbA1c level (median: 11.30% (7.31 to 16.00) vs. 9.30% (4.40 to17.31), *p*=0.022).

**Conclusion:**

The current study gave evidence of a high prevalence of CD specific auto-antibodies in T1D population. The co-existence of these two conditions was associated with a poor glycemic control, a lower height, and other autoimmune diseases. These findings may suggest the necessity of a systematic screening of CD in T1D patients.

## 1. Introduction

The association between celiac disease (CD) and type 1 diabetes (T1D) was first reported during the sixties [1]. This association has become nowadays well-known and more common with the development of sensitive and specific related serological tests.

The prevalence of CD in T1D patients is reported to be 5 to 7 times greater than in the general population [2]. In a meta-analysis gathering 27 studies with a total of 26,605 T1D patients, Elfström found that the overall prevalence of biopsy-proven CD is almost 6% [3]. Till now, there are few relevant published data about the prevalence of CD in the Moroccan general population and also in common clinical conditions usually associated with CD such as diabetes or autoimmune thyroiditis (AIT) [4, 5].

Both diseases share some common genetic background, mainly HLA molecules. In fact, the HLA-DQ2 genotype is found in 90% of celiac patients and in 55% of diabetics [6]. It was also reported that 33% of T1D patients who are homozygous for HLA-DQ2 are seropositive for anti-tTGA (tissue transglutaminase antibodies) versus 2% who are negative for HLA-DQ2/8 [7]. On the other hand, the role of gluten [8] and viral infections [9, 10] in the etiopathogenesis of T1D is still a matter of controversy. Viral infections were also investigated in CD [11].

The presence of CD characterizes a subgroup of T1D patients that displays potential high risks of hypoglycemia [12, 13], microvascular complications [14, 15], and overall mortality [16]. Therefore, most of the guidelines recommend a systematic screening for CD in TID [17–19].

The main objective of the study was to determine the prevalence of specific auto-antibodies to CD in T1D in a Moroccan adult and pediatric population and to describe the clinical and biological characteristics of seropositive diabetic patients.

## 2. Patients and Methods

### 2.1. Study Design

A multicenter cross-sectional study was conducted in 276 adult and pediatric diabetic patients who were enrolled from five centers, including 2 pediatric centers, 2 adult endocrinology centers, and 1 specialized primary health care center. Patients were randomly recruited over a period of 23 months.

The sample size of the study was calculated with an estimated precision of the prevalence equal to 3% with a confidence interval of 95%.

All patients were interviewed by the same investigator using a hetero-administered questionnaire focusing on most relevant symptoms of CD such as abdominal pain, bloating, diarrhea, constipation, vomiting, chronic fatigue, anorexia, and pallor, history of bone fractures and recent weight loss, ketoacidosis episodes, and hypoglycemic events that had occurred over a period of 6 months before the enrollment.

Patients' blood samples were collected and stored at −20°C for subsequent serial testing.

The samples were tested for 4 relevant associated T1D auto-antibodies, including anti-GAD65-Ab (Aeskulisa™ GAD65, GmbH, threshold: 30 IU/ml), anti-Insulin-Ab (Aeskulisa™ Insulin, GmbH, threshold: 18 IU/ml), anti-IA2-Ab (Aeskulisa™ IA2, GmbH, threshold: 30 IU/ml), and anti-ZnT8-Ab (Aeskulisa™ anti-ZnT8, GmbH, threshold: 15 IU/ml).

According to ESPGHAN guidelines for the diagnosis of CD [17], patients underwent an immunologic testing initially based on IgA anti-tTGA screening combined with IgA subclass quantification. Only positive IgA-tTGA cases were investigated for IgA anti-endomysial antibodies (EMA), and those who displayed low IgA-tTGA titers (<10 times the cutoff value) were proposed for HLA DQ2/DQ8 typing. The IgA deficiency cases underwent IgG anti-tTGA testing.

### 2.2. Laboratory Testing Methods

- Anti-tTG IgA and IgG testing was performed using commercial ELISA kits (tTG ELISA Orgentec, Diagnostika GmbH, threshold: 10 IU/ml).
- EMA testing was done by an indirect immunofluorescence assay (Euroimmun AG, Lübeck, threshold: 1 : 10), with a semiquantitative assessment.
- IgA class dosage was evaluated using a nephelometry system (BN Prospec, Siemens).
- HLA DQ2 and DQ8 typing were performed on the basis of PCR-SSP (polymerase chain reaction-sequence specific primers) technique (One Lambda, USA) realized on DNA extracted from patient samples. Positive tTGA and EMA testings or irresolute cases following the serology and HLA typing were recommended for intestinal biopsy to confirm or to rule out the diagnosis of CD.

### 2.3. Statistical Analysis

The data were recorded and then analyzed using SPSS 16.0 statistical software. Descriptive statistics (mean, standard deviations, median, range, and proportions) were used to represent the trend and distribution of the results. The comparison between the 2 subgroups (celiac and diabetics vs. diabetics) was made using the independent *T* test and the Mann–Whitney *U* test for continuous outcomes and by chi-squared test and Fisher's exact test for proportions. Results were considered statistically significant when *p* value is less than 0.05.

### 2.4. Ethical Considerations

According to the Declaration of Helsinki (1964), patients have been informed about the objectives of the study. An informed consent was obtained from patients or their tutors if minors, prior to enrollment.

## 3. Results

The mean age of patients was 14.1 ± 8 years, ranging from 1 to 42 years, with 144 females versus 132 males (sex-ratio M/F = 0.91). The mean duration of diabetes was 5.4 ± 5.3 years.

The overall seroprevalence of CD markers was 9.1% (IC = 95%) including 24 positive cases of IgA-tTGA and 1 positive case of IgG-tTGA that displayed an IgA deficiency (seronegative for IgA-tTGA). Among all positive cases, 17 of them had high titers of tTG antibodies (>100 IU/ml) and 8 others had titers ranging between 13.23 and 68 IU/ml. All the 24 positive cases on IgA-tTGA were also positive on EMA testing. The other one had IgA deficiency and had not been tested for IgA-EMA.

Among 8 cases of low IgA-tTGA titers, 4 were positive for HLADQ2, 1 was positive for HLADQ8, and 1 was positive for both HLADQ2 and DQ8. The two other cases, including the IgA deficiency case, had undergone an intestinal biopsy, confirming CD and went on GFD (Table 1).

The mean age of the pediatric population was 9 ± 3.7 years, of which, 17 were seropositive for CD specific markers. In contrast, the adult population had a mean age of 21 ± 6 years, and 8 of them were seropositive as well. The other demographic, clinical, and biological characteristics of the two populations are shown in Table 2.

Among the associated T1D-tested auto-antibodies, 79.7% of cases had at least one positive auto-Ab, 42.4% had positive anti-GAD65 Ab, 36.2% had positive anti-ZnT8 Ab, 31.9% had positive anti-Insulin Ab, and 28% had anti-IA2 Ab. The frequency of auto-Ab categories is given in Tables 3 and 4, according to the population groups and CD-specific auto-Ab status, respectively.

According to the age at the onset of diabetes, the autoimmunity status of our patients (positive versus negative) was as follows: 63.2% vs. 67.9%, 29.5% vs. 21.4%, 6.4% vs. 10.7%, and 0.9% vs. 0% in less than 10 years, between 10 and 20 years, between 20 and 30 years, and superior to 30 years, respectively.

Clinically, we observed a significant statistical difference between seropositive and seronegative cases concerning pallor (32% vs. 9.2%; *p*=0.003), chronic constipation (56% vs. 27.5%; *p*=0.003), abdominal pain (72% vs. 37.8%; *p*=0.001), bloating (64% vs. 30.7%; *p*=0.001), and recent weight loss (68% vs. 45.4%; *p*=0.031). No statistically significant differences were noticed about diarrhea, chronic fatigue, loss of appetite, and vomiting (Table 4). Globally, CD seropositivity was significantly associated with a higher proportion of other autoimmune diseases (16% vs. 2.4%, *p*=0.008). In the seropositive group, we noticed 1 case of autoimmune thyroiditis, 1 case of Addison's disease, 1 case of lupus, and 1 case of idiopathic juvenile arthritis, versus 3 cases of autoimmune thyroiditis, 2 cases of Biermer anemia, and 1 case of Addison's disease in the seronegative group.

Seropositive patients had a higher median of HbA1c (11.30 (7.31 _ 16.00) vs 9.30 (4.40 _ 17.31); *p*=0.022) and a lower *Z* score of height (−0.90 (−3.93 _ 0.95) vs −0.51 (−4.54 _ 2.18); *p*=0.029).

We observed that seropositive patients displayed a tendency towards a higher percentage of history of bone fractures (20% vs. 8.8%, *p*=0.081), a higher mean insulin dosage (0.91 UI/Kg ± 0.34 vs. 0.80 UI/Kg ± 0.27; *p*=0.064), a higher female percentage (68% vs. 50.6%; *p*=0.097), and a higher frequency of bad glycemic control (78.3% vs. 57.8%, *p*=0.056).

No significant differences were observed between the seropositive and seronegative groups regarding the mean age (12.95 years ± 8 vs. 14.2 years ± 8; *p*=0.763), the mean diabetes duration (6.8 years ± 7.5 vs. 5.2 years ± 5.1; *p*=0.295), and the mean age at diabetes onset (6.8 years ± 4.7 vs. 8.9 years ± 6.4; *p*=0.113). There was no significant difference in relation to the overall percentage of familial cases of autoimmune diseases (40% vs. 38.2%, *p*=0.864), the consanguinity rate (24% vs. 20%; *p*=0.636), the median Z-scores of both weight (−0.50 (−3.35 _ 1.48) vs. −0.33 (−3.83 _ 3.00); *p*=0.216), and body mass index (BMI) (−0.07 (−2.50 _ 1.24) vs. −0.04 (−4.87 _ 3.43); *p*=0.880) and the frequency of ketoacidosis at the onset of T1D (66.7% vs. 60.9%, *p*=0.58) or hypoglycemic events between the two groups.

## 4. Discussion

Our results display a relatively high prevalence of CD-specific auto-antibodies in Moroccan T1D patients. Actually, a similar Moroccan study has shown a higher rate of tTGA positivity (13%), but regarding the very small sample of adult T1D patients studied (31 cases), these data seem to overestimate the real prevalence of associated auto-antibodies to CD [4]. In contrary and in a similar context, Azzi et al. [5] reviewed the records of 720 T1D pediatric patients and concluded a biopsy-proven CD prevalence of 3.05%. However, the authors have considered this rate to be a definite underestimation since most of patients were not systematically screened for CD. On the other hand, the prevalence rate found in our study is comparable to that reported by Barerra et al. (9.9%), Salardi et al. (8.8%), Larsson et al. (9%), Gabriel et al. (9.2%), Gillet et al. (8.2%), and Mankaï et al. (8.3%) [20–25]. However, it is much higher than that reported by Mont-Serrat et al. (2.5%) [26] and lower than the findings of Boudraa et al. (16.4%), Al-Hussaini et al. (20%), and Ashabani et al. (21.3%) [27–29]. In fact, the reasons behind the variability of the prevalence rate of CD in T1D patients between different countries (Table 5) remain mostly unexplained [3]. On the other side, the potential impact of sex and age on the frequency of CD was assessed by Cerutti et al. and concluded in a large cohort of 4322 T1D and CD patients that female sex is an independent predictive risk factor for developing CD [33]. Similar findings have also been reported by many other authors [27, 34–38]. However, Pham-short et al. did not find such associations in their large meta-analysis gathering 26 studies [39].

Our study did not display a significant difference in the mean age between the 2 groups of patients as many similar studies did [27, 31, 34, 40, 41]. However, Cerutti et al. reported that the onset of diabetes before 4 years of age is an independent risk factor for developing CD (OR: 3.27 95% IC 2.20–4.85) [33]. Such observation is also supported by many other authors [25, 31, 34, 36, 40, 42]. The results concerning the duration of diabetes remain, however, more controversial probably due to differences in CD screening strategies as well as the design of the studies.

We observed significant differences regarding the following clinical findings: pallor, chronic constipation, abdominal pain, bloating, and recent weight loss. These findings are comparable to those of the study of Hansen et al. [31] that displayed significant differences between 33 CD-T1D patients compared to 236 T1D only, concerning abdominal pain, loose and/or frequent stool, bloating, constipation, arthralgia, tiredness, and frequent hypoglycemia, whereas no difference was reported about other symptoms like aphthous ulcers and tooth enamel defects.

On another side, the impact of CD on growth in diabetic patients as well as the potential benefit of the gluten-free diet (GFD) on this parameter is still controversial. About that, Hansen et al. [31] found that at the time of CD diagnosis, CD-T1D patients had a smaller mean weight SD than T1D patients. Other authors reported similar results for weight SD [27, 30, 34, 43] and height SD [20, 30, 34, 43]. Our study showed a significant difference between the two groups in only height SD.

It is known that CD is generally associated with an increased risk of having other autoimmune diseases (AID). By the way, Not et al. [44] reported a strong association of CD with the presence of other AID in T1D patients (37.5% vs. 6.3%, *p* < 0.0001). The most frequent AID in such a population is AIT [31, 33]. The overall percentage of AID among seropositive patients in our series is high and statistically significant (16%) but might be underestimated, because there was no systematic screening for AID in both diabetic and celiac patients. Actually, the seroconversion of CD happens in the first years of the T1D onset and almost 85% are diagnosed by 5 years [45].

Furthermore, it is recommended to screen for CD in T1D patients who particularly manifest unexplained bad glycemic control [46]. This recommendation is based on the study of Leeds et al. [13] who observed that CD-T1D patients have a higher HbA1c level in comparison with T1D adult patients (8.2% vs. 7.5%, *p*=0.05). Similarly, our study confirms these findings and shows a significantly elevated median of HbA1c level in seropositive cases, in contrary to several series showing no significant difference [23, 27, 28, 31, 35, 47–49].

On the basis of the potential impact of CD on insulin requirements, some authors recommend a screening for CD in patients with unusual low doses of insulin. In fact, this may be explained essentially by malabsorption. Linked to that, a case-control study conducted by Mohn et al. noticed the reduction of insulin doses 12 months prior to CD diagnosis (0.6 UI/Kg vs. 0.9 UI/Kg, *p*=0.05). The same observation was reported by Abid et al. and Poulain et al. [36, 50]. The authors reported a rise in insulin requirements after the startup of a GFD [12, 51]. Regarding our cases, we observed a tendency towards higher insulin doses in seropositive patients, which may reflect the differences in glycemic control for this group of patients.

T1D is associated with a 2- to 4-fold increase of fractures' global risk [52]. To the same extent, Heikkilä et al. has found in their meta-analysis that CD is associated with an increase in the global risk of fractures with an OR of 1.30 [53]. Furthermore, Lunt et al. demonstrated that BMD (bone mineral density) evaluated by DXA (dual-energy X-ray absorptiometry) in the lumbar region of nontreated adult celiac and type 1 diabetic women is inferior in comparison with type 1 diabetics only [54]. In our patient series, we observed a higher proportion of positive bone fractures' history in T1D patients compared to seronegative diabetics (20% vs. 8.8%, *p*=0.081). This observation requires further investigation especially in the adult population.

On another side, the influence of CD status on the course of T1D remains controversial, especially regarding degenerative complications [55]. However, a Swedish study pinpointed the fact that it is the duration of CD that conditions the impact of CD on diabetic retinopathy [15]. The authors reported a beneficial effect the first 5 years (HR 0.57 95% CI: 0.36–0.91), no effect during the following 5 years (HR 1.03 95% CI: 0.68–1.57) and an aggravation after 10 years (HR of 2.83 after 10 years). Otherwise, CD is associated with a higher risk of end-stage renal disease in T1D patients, with a HR of 2.03 after 10 years [55]. Leed et al. have reported similar results concerning diabetic nephropathy, with a positive effect of GFD on renal markers after 1 year [13].

The effect of CD status on the severity of T1D onset has been studied by Rami et al. [49] and did not report any association. Our study came to the same conclusions. However, a clinical form of CD occurring before T1D has been reported in the literature, characterized by severe diabetes onset and a higher prevalence of other AID [56]. Similarly, our only patient for whom the diagnosis of CD was made before T1D onset corresponded to a case of IgA deficiency associated with epilepsy, with Addison's disease and asthma. Moreover, CD does not seem to be associated with a higher risk of ketoacidosis and diabetic coma during the course of T1D [30, 49, 57, 58].

To the best of our knowledge, our study is the first relevant multicenter cross-sectional study conducted in Morocco. At the end, our results give evidence of a high CD-specific auto-antibodies positivity in T1D but remain relatively limited to well assess the impact of such associations on the course of T1D. In fact, the small number of seropositive patients and the necessity of a further intestinal biopsy as well as a follow-up are the factors to be considered in the conclusions to be drawn from these data. With the same perspectives in mind, we have communicated the findings of our CD laboratory testing to the physicians for further investigation (biopsy) and GFD if required, with an active surveillance.

## 5. Conclusion

The current study concluded a high prevalence of CD-specific antibodies in Moroccan T1D patients. In comparison with the diabetic group, seropositive celiac and diabetic patients displayed significant association with poor glycemic control, lower height SD, and an increased frequency of other AIDs.

These findings emphasize the benefit of a systematic screening for CD in diabetic patients, which can hopefully minimize the potential risk of complications due to undiagnosed CD. Furthermore, controlled longitudinal studies in a larger sample with an assessment of the potential benefit of a GFD, especially in asymptomatic patients, are needed.

## Figures and Tables

**Table 1:** The immunological profile of seropositive celiac patients.

Patients	Age (yrs)	Anti-tTG IgA (IU/ml)	Anti-EMA IgA intensity	Anti-tTG IgG (IU/ml)	IgA dosage	HLA DQ2/8 typing
Patient 01	2	68	++	−	NL	DQ8
Patient 02	2	>200	++++	−	NL	−
Patient 03	3	31	++	−	NL	DQ2
Patient 04	7	>200	+++	−	NL	−
Patient 05	7	>200	++++	−	NL	−
Patient 06	8	130	+++	−	NL	−
Patient 07	9	>200	++++	−	NL	−
Patient 08	10	>200	++++	−	NL	−
Patient 09	10	>200	+++++	−	NL	−
Patient 10	10	>200	++++	−	NL	−
Patient 11	10	>200	++++	−	NL	−
Patient 12	11	>200	++++	−	NL	−
Patient 13^*∗*^	11	37	+	−	NL	−
Patient 14	12	>200	++++	−	NL	−
Patient 15	13	>200	+++	−	NL	−
Patient 16	14	13.23	+	−	NL	DQ2
Patient 17	14	112	++	−	NL	−
Patient 18	15	45	+	−	NL	DQ2
Patient 19	15	58	++	−	NL	DQ2
Patient 20	15	45	+	−	NL	DQ2/DQ8
Patient 21^*∗*^	16	NG	−	85	<0.26 g/L	−
Patient 22	18	125	++++	−	NL	−
Patient 23	23	>200	++++	−	NL	−
Patient 24	33	>200	++++	−	NL	−
Patient 25	35	>200	++++	−	NL	−

^*∗*^Patient was confirmed CD with small bowel biopsy; NG = negative; NL = normal.

**Table 2:** Demographic, clinical, and biological characteristics of the pediatric and adult populations of our series.

	Age (Y)	Sex	Height (SD)	Weight (SD)	BMI (SD)	T1D family history	CD family history	HbA1c (%)	Age at T1D onset (Y)	T1D duration (Y)	Daily insulin dose (IU/Kg)	Known microvascular complications	Known macrovascular complications	Insulin type prescribed	Insulin regimen	Severe hypoglycemia episodes
Children <15 years (*n* = 167)	9 ± 3.7	48.5% F	−0.57 ± 1.2	−0.36 ± 1.1	−0.13 ± 1.3	19.2%	4.2%	9.6 ± 2.4	6 ± 3.5	3.1 ± 2.9	0.75 ± 0.2	0%	0%	HI (89%) AN (10.8%)	2 INJ (91.6%) BB (7.8%) 3 INJ (0.6%)	<1/M (93.4%) 1–4/M (6.6%)
Adults ≥15 years (*n* = 109)	21 ± 6.6	57.8% F	NA	NA	NA	24.8%	5.5%	10.1 ± 2.7	12 ± 7.3	8.8 ± 6.2	0.9 ± 0.3	12.8%	1.8%	HI (77.1%) AN (21.1%)	2 INJ (49.5%) 3 INJ (27.5%) BB (11.9%) FI (11%)	<1/M (95.4%) 1–4/M (1.8%) >1/W (2.8%)

HI: human insulin; AN: analogues; INJ: injections; BB: basal bolus; FI: functional insulin therapy; M: month; W: week; NA: not applicable.

**Table 3:** The frequency of associated T1D auto-antibodies in the pediatric and adult populations of our series.

	Overall positivity of T1D markers, *n* (%)	GAD65-Ab, *n* (%)	Anti-insulin Ab *n* (%)	IA2-Abs *n* (%)	ZnT8-Ab*n* (%)	Positivity of specific CD Abs *n* (%)
Children <15 y (*n* = 167)	(145) 86.8%	(77) 46.1%	(51) 30.5%	(61) 36.5%	(69) 41.3%	(17) 10.2%
Adults ≥15 y (*n* = 109)	(75) 68.8%	(40) 36.7%	(37) 33.9%	(17) 15.6%	(31) 28.4%	(8) 7.3%

**Table 4:** Comparison of clinical and biological profiles between seropositive and seronegative patients.

	Seropositive group, *n* (%)	Seronegative group, *n* (%)	*p* value
Clinical manifestations			
Pallor	8 (32%)	23 (9.2%)	**0.003** ^*∗*^
Chronic constipation	14 (56%)	69 (27.5%)	**0.003** ^*∗*^
Abdominal pain	18 (72%)	94 (37.8%)	**0.001** ^*∗*^
Bloating	16 (64%)	77 (30.7%)	**0.001** ^*∗*^
Recent weight loss	17 (68%)	114 (45.4%)	**0.031** ^*∗*^
Diarrhea	9 (36%)	54 (21.9%)	0.111
Nausea and vomiting	4 (16%)	31 (12.7%)	0.416
Chronic fatigue	13 (52%)	92 (36.7%)	0.132
Lack of appetite	9 (36%)	70 (27.9%)	0.392
Other associated AIDs	4 (16%)	6 (2.4%)	**0.008** ^*∗*^
Median *Z* scores of height	−0.90 (−3.93 _ 0.95)	−0.51 (−4.54 _ 2.18)	**0.029** ^*∗*^
Median *Z* scores of BMI	−0.07 (−2.50 _ 1.24)	−0.04 (−4.87 _ 3.43)	0.880
Median HbA1c	11.30 (7.31_16.00)	9.30 (4.40 _ 17.31)	**0.022** ^*∗*^
T1D-tested auto-antibodies			
GAD65-ab (%)	(13) 52%	(104) 41.4%	0.308
GAD65-ab mean titers (UI/ml)	231.1 (33.15–500)	184.2 (30.02–500)	0.096
Anti-insulin-ab (%)	(7) 28%	(81) 32.3%	0.662
Anti-insulin-ab mean titers (UI/ml)	83.49 (18.04–300)	47.99 (18.24–300)	0.603
IA2-ab (%)	(11) 44%	(67) 26.7%	0.067
IA2-ab mean titers (UI/ml)	109.8 (30.85–370.5)	176.54 (30.29–500)	0.064
ZnT8-ab (%)	(11) 44%	(89) 35.5%	0.397
ZnT8-ab mean titers (UI/ml)	170.77 (16–47–500)	194.43 (15.10–500)	**0.002** ^*∗*^
Overall positivity (%)	(23) 92%	(197) 78.5%	0.109

AID: autoimmune diseases; BMI: body mass index. ^*∗*^Statistically significant differences.

**Table 5:** Seroprevalence rate of CD in T1D according to different series.

Author	Country	Sample size	Mean age	Seroprevalence rate
Europe				
Crone et al. [30]	Austria	157	14.8 (4–21)	13.5%
Hansen et al. [31]	Denmark	269	10.9 (1.5–16)	12.1%
Barerra et al. [20]	Italy	273	—	9.9%
Salardi et al. [21]	Italy	331	8.1 ± 4.3	8.8%
Larsson et al. [22]	Sweden	300	—	9.0%

North America				
Aktay et al. [32]	USA	218	13.7 (4–21)	7.7%
Gillet et al. [24]	Canada	233	12.9 (1.3–19.2)	8.2%

Middle east and north Africa				
Bouderra et al. [29]	Algeria	116	(1–19.5)	16.4%
Al-Hussaini et al. [27]	Saudi Arabia	106	8.5 (1–15.5)	20%
Ashabani et al. [28]	Libya	234	12.8 (2–50)	21.3%
Mankai et al. [25]	Tunisia	205	11 (1–11)	2.5%
Our study	Morocco	276	14.1 (1–42)	9.1%

## Data Availability

The data used to support the findings of this study are available from the corresponding author upon request.
